# Is Gastrectomy-Induced High Turnover of Bone with Hyperosteoidosis and Increase of Mineralization a Typical Osteomalacia?

**DOI:** 10.1371/journal.pone.0065685

**Published:** 2013-06-11

**Authors:** Takashi Ueyama, Yuta Yamamoto, Kazuki Ueda, Aiji Yajima, Yoshimasa Maeda, Yasunobu Yamashita, Takao Ito, Yoshihiro Tsuruo, Masao Ichinose

**Affiliations:** 1 Department of Anatomy and Cell Biology, Wakayama Medical University Graduate School of Medicine, Wakayama, Japan; 2 2nd Department of Internal Medicine, Wakayama Medical University Graduate School of Medicine, Wakayama, Japan; 3 Department of Pathology, Kuro-òs lab, The University of Texas, Southwestern Medical Center at Dallas, Dallas, Texas, United States of America; 4 Division of Renal Replacement Therapeutic Science, Akita University School of Medicine, Akita, Japan; Oklahoma State University, United States of America

## Abstract

Gastrectomy (GX) is thought to result in osteomalacia due to deficiencies in Vitamin D and Ca. Using a GX rat model, we showed that GX induced high turnover of bone with hyperosteoidosis, prominent increase of mineralization and increased mRNA expression of both osteoclast-derived tartrate-resistant acid phosphatase 5b and osteocalcin. The increased 1, 25(OH)_2_D_3_ level and unchanged PTH and calcitonin levels suggested that conventional bone and Ca metabolic pathways were not involved or changed in compensation. Thus, GX-induced bone pathology was different from a typical osteomalacia. Gene expression profiles through microarray analysis and data mining using Ingenuity Pathway Analysis indicated that 612 genes were up-regulated and 1,097 genes were down-regulated in the GX bone. These genes were related functionally to connective tissue development, skeletal and muscular system development and function, Ca signaling and the role of osteoblasts, osteoclasts and chondrocytes. Network analysis indicated 9 genes (Aldehyde dehydrogenase 1 family, member A1; Aquaporin 9; Interleukin 1 receptor accessory protein; Very low density lipoprotein receptor; Periostin, osteoblast specific factor; Aggrecan; Gremlin 1; Angiopoietin-like 4; Wingless-type MMTV integration site family, member 10B) were hubs connected with tissue development and immunological diseases. These results suggest that chronic systemic inflammation might underlie the GX-induced pathological changes in bone.

## Introduction

Gastrectomy (GX) reduces bone mineral content in humans [1]–[3], and the condition has been designated as osteomalacia due to deficiencies in vitamin D (VD) and calcium (Ca) as described in textbooks of orthopedics and endocrinology. Accordingly, studies of GX-induced bone pathology have not been investigated further. The aims of this study were to re-evaluate the histological, biochemical and molecular changes in GX-induced bone using animal models and more advanced procedures.

These conditions can be reproduced in rats by GX or fundectomy (FX), the resection of the acid-producing part of the stomach [4]–[6]. GX or FX in rats induced a marked and rapid reduction in bone mineral density and trabecular bone volume [4]. Subcutaneous infusion of Ca did not prevent the bone loss after GX, suggesting that the consequences of GX are not related to Ca mal-absorption or Ca deficiency [4], [5]. Others demonstrated that GX-induced osteopenia was not due to a deficiency of vitamin B_12_, folic acid or Ca, and it was independent of the type of anatomic reconstruction of the digestive tract [6]. Treatment with a proton pump inhibitor (PPI) had no effects on the decrease in bone mineral density, suggesting that lack of gastric acid seemed not to contribute to the bone loss after GX [4]. However, the gastric fundus, the acid-producing part of the stomach was critical for bone metabolism [7]–[9], since osteopenia could be preserved by retaining 10∼30% of the fundic mucosa [8].

Detailed biochemical analysis in response to FX [9] showed increased levels of urinary excretion of phosphorus (P) and cAMP, decreased level of urinary pH, normal levels of serum parathyroid hormone (PTH), calcitonin, 25(OH)D_3_, Ca, magnesium (Mg), and inorganic phosphate (iP), and increased levels of serum gastrin and 1, 25(OH)_2_D_3_. Following oral Ca challenge, thyroid-intact FX rats showed hypercalcemia, decreased levels of serum gastrin, increased level of calcitonin and decreased level of PTH. Thyroidectomized FX rats showed hypercalcemia, normal level of calcitonin, and decreased level of PTH. The serum levels of gastrin were not correlated with the levels of calcitonin or PTH, and the only predictor of serum 1, 25(OH)_2_D_3_ was urinary phosphorus in multivariate regression analysis. The authors concluded that in the FX rats (1) osteopenia was not caused by intestinal Ca mal-absorption, VD, Ca deficiency, or secondary hyperparathyroidism; (2) osteopenia might be related to PTH-independent urinary hyperexcretion of P, followed by a rise of serum 1, 25(OH)_2_D_3_; and (3) the existence of endocrine axis among gastrin, calcitonin, and PTH could not be substantiated. GX-induced osteopenia was considered to be due to stimulated bone resorption rather than to reduced bone formation since GX had no effect on the bone regeneration process in artificial transosseous defects produced in the mandible [10].

Recently, GX has been used less often in clinical settings due to early detection of gastric cancer and less invasive treatments instead of GX. On the contrary, atrophic gastritis associated with Helicobacter pylori infection [11], [12] or long-term treatment with PPI [13], [14] was associated with increased risk of osteoporosis and fracture, although short-term treatment with PPI in rats had no effects on bone [4]. The dosages and duration of PPI used in animal studies [4], [15] attained an intra-gastric pH of over 4.0 [15]. In contrast, GX or atrophic gastritis, and longer-term treatment with PPI resulted in neutral pH in the gastro-intestinal tract. Persistent neutral pH may result in pathological bone changes.

Research studies in GX-induced animal models are very limited despite a lack of understanding of its pathogenesis. In this study, we have re-evaluated the pathological characteristics of the GX-induced rat model using more advanced technologies. First, we evaluated the bone mineral density using X-ray computed microtomography (CT) scanning. Second, bone histomorphometry was used for evaluating the precise bone structural and kinetic changes. Third, serum contents of factors and hormones related with bone and Ca metabolism were estimated. Fourth, mRNA expression involved in bone and Ca metabolism was estimated by quantitative real-time RT-PCR. These results revealed reduced bone mineral density, prominently increased mineralization, high turnover of bone with hyperosteoidosis, and increased mRNA expression of both osteoclast-derived tartrate-resistant acid phosphatase 5b and osteocalcin. Increased 1, 25(OH)_2_D_3_ levels and unchanged PTH and calcitonin levels suggested that conventional bone and Ca metabolic pathways were not involved or changed in compensation. A new and important finding of this study was to challenge the consensus that GX-induced bone is a typical osteomalacia.

Finally, we assessed the GX-induced alteration of gene expression profiles in bone using microarray analysis and we explored the functional pathways and the networks using Ingenuity Pathway Analysis (IPA). We found that the GX-induced high turnover of bone with hyperosteoidosis and the increased mineralization were associated with an alteration in the expression of thousands of genes in bone, and that these were functionally related to connective tissue development, skeletal and muscular system development and function, Ca signaling and the role of osteoblasts, osteoclasts and chondrocytes. Network analyses indicated 9 genes (Aldehyde dehydrogenase 1 family, member A1; Aquaporin 9; Interleukin 1 receptor accessory protein; Very low density lipoprotein receptor; Periostin, osteoblast specific factor; Aggrecan; Gremlin 1; Angiopoietin-like 4; and Wingless-type MMTV integration site family, member 10B) were the hubs connected with tissue development and immunological diseases. Another new and interesting outcome of this study is the suggestion that chronic systemic inflammation may underlie the pathogenesis of GX-induced bone changes, the precise mechanisms of which are yet to be elucidated.

## Materials and Methods

### Surgery

Total gastrectomy (GX, 10 week-old male Wistar rats, n = 8) was performed by resecting the stomach followed by anastomosing the duodenum and esophagus end-to-end under aseptic conditions. Medetomidine hydrochloride (0.15 mg/kg), midazolam (4 mg/kg) and butorphanol tartrate (5 mg/kg) as anesthesia produced the desired levels of sedation, analgesia, amnesia, and skeletal muscle relaxation. Postoperatively, the animals were treated with subcutaneous infusion of Solita®-T3 (Ajinomoto, Tokyo, Japan) to prevent dehydration. Sham operation (n = 6) consisted of manipulation of the viscera, and these animals were used as controls. The mortality of total gastrectomy was 25%. The rats were provided a commercial powdered diet (MF, Oriental Yeast Co., Tokyo, Japan) starting on the day following surgery. The Ca concentration of the diet was 1.12 g/100 g. Body weight of each rat was measured daily. The body weights of sham-operated rats were adjusted to those of GX rats by controlling their diets. Five weeks after surgery, under anesthesia with medetomidine hydrochloride (0.15 mg/kg), midazolam (4 mg/kg) and butorphanol tartrate (5 mg/kg), blood was collected from the right atrium and about 3 ml of serum was obtained. Tissue samples were collected immediately from the liver, kidney, thyroid, pituitary gland and femur. The left femur, after removal of the muscles and connective tissues, were fixed with 4% paraformaldehyde in 0.1 M phosphate buffer, pH 7.4 for 2 days at 4°C, then stored in phosphate-buffered saline (PBS) containing 30% sucrose at 4°C. The liver, kidney, thyroid, pituitary gland, and right femur were stored at −80°C in a RNase stabilizing solution (RNA later® solution, Ambion, USA).

All procedures were approved by the Wakayama Medical University Animal Care and Use Committee.

### X-ray Computed Microtomography (CT) Scanning

Fixed femurs were subjected to X-ray microtomography using a computed-tomography apparatus for small experimental animals (Model LaTheta LCT-200; Hitachi-Aloka, Tokyo, Japan) [16]. Continuous 96 µm slice images were utilized for quantitative assessments using the LaTheta software (version 3.20). Bone mineral content (milligrams), bone volume (cubic centimeters), and bone mineral density (milligrams per cubic centimeters) were calculated according to the protocol provided by Hitachi-Aloka (see in details, http://www.hitachi-aloka.co.jp/products/data/animal-001-LCT-200). The minimum moment of inertia of cross-sectional areas (milligram-centimeters), which represents the flexural rigidity, and the polar moment of inertia of cross-sectional areas (milligram-centimeters), which represents the torsional rigidity were also calculated automatically by the LaTheta software.

### Bone Histomorphometry

Other groups of rats were gastrectomized (10 week-old male Wistar rats, n = 8) or sham-operated (n = 6) as described above. The mortality of total gastrectomy was 25%. For bone histomorphometrical analysis, these rats were double-labeled with subcutaneous injections of 20 mg/kg tetracycline hydrochloride (Sigma, St. Louis, MO, USA) for 168 h and 10 mg/kg calcein (Dojindo, Kumamoto, Japan) for 48 h before sampling. Femurs and calvariae were removed from each rat and fixed with 70% ethanol. They were trimmed to remove muscle, stained with Villanueva bone stain for 5 days, dehydrated in graded concentrations of ethanol and embedded in methylmethacrylate (Wako Chemicals, Kanagawa, Japan) without decalcification. Frontal plane sections (5 µm) of the femoral diaphysis and calvaria, and sagittal plane sections (5 µm) of the femoral distal epiphysis were cut using a microtome (Lieca, Germany). The cancellous bone was measured in the secondary spongiosa.

The primary parameters of bone structure were measured in the femoral distal epiphysis and the secondary parameters including bone volume, erosion, formation and mineralization were calculated according to published standards [17]–[19].

### Analysis of Serum Samples

Serum Ca concentrations were measured by the o-cresolphthalein complexone method (Nescoat® Ca-V2, Alfresa, Tokyo, Japan). Serum iP concentrations were measured by the maltosephospholylase method (Determina L® IP II, Kyowa Medex, Tokyo, Japan). Serum 1, 25(OH)_2_D_3_ concentrations were measured by RIA kit (TFB, Tokyo, Japan). Serum 25(OH)D_3_ concentrations were measured by RIA kit (DiaSorin, MN, USA). Serum PTH concentrations were measured by ELISA kit specific for rat PTH (Uscn, Wuhan, China). Serum calcitonin concentrations were measured by RIA kit specific for human calcitonin with rat calcitonin as the standard (the cross reactivity is 65%) (Mitsubishi Chemical Medicine, Tokyo, Japan).

### Quantitative Real-time RT-PCR

Total RNAs from the liver, kidney, thyroid and pituitary gland were extracted using RNeasy® Mini Kit (QIAGEN, Tokyo, Japan). After removal of connective tissue and bone marrow, femoral diaphyses were quickly frozen in N_2_ liquid and powdered in an earthenware mortar. Total RNAs from powdered bone were extracted by RNeasy® Lipid tissue Mini Kit (QIAGEN, Tokyo, Japan). The analysis of RNA quality showed that the 260∶280 nm absorbance ratio of RNA samples used in this experiment ranged consistently from 1.8 to 2.0. The qualities of purified RNAs were assessed by an Agilent 2100 Bioanalyzer using an RNA 6000 Nano Kit (Agilent Technologies, Palo Alto, CA, USA).

Expression of each mRNA was determined by real-time RT- PCR. Primer sets for each gene are listed in Table 1. As an internal control, we also estimated the expression of rat glyceraldehyde-3 phosphate dehydrogenase (GAPDH) mRNA. Total RNA (0.1 µg) was converted into cDNA by reverse transcription using random primer p (dN)_ 6_ primers and AMV reverse transcriptase (Roche Diagnostics Corp., Indianapolis, IN, USA) in a total reaction volume of 20 µl. PCR amplification using a LightCycler instrument was carried out in 20 µl of reaction mixture consisting of LightCycler FastStart DNA Master SYBR Green I (Roche Diagnostics GmbH, Penzberg, Germany), 4.0 mM MgCl_2_, 0.5 µM of each probe, and 2 µl of template cDNA in a LightCycler capillary. Relative mRNA in each sample was then quantified automatically by reference to the standard curve constructed each time according to the LightCycler software. The levels of mRNA were calculated with reference to external standard curves constructed by plotting the log number of 10-fold serial diluted cDNA samples against the respective threshold cycle with second derivative maximum method. Expression of mRNA level in each sample was normalized against its GAPDH mRNA level.

**Table 1:** List of oligonucleotide primers used for RT-PCR.

Gene	Accession number	Forward primer	Reverse primer
Osteoclast-derived Tartrate-resistant Acid phosphatase 5b	NM_019144	CCATTGTTGGCTGCATAC	AGAAGGGTCCATGAAGTTG
Osteocalcin	NM_001033860	GTCTGTGCAATTTCGTGTA	CAGGTAGCTGGTATTGTTAGTGT
RANK	NM_012870	AGAGAGGATAAAACGGAGACA	TGCTTTCACAGAGGTCAAT
RANKL	NM_057149	TCAGGAGTTCCAGCTATGAT	CAAGAGGACAGACTGACTTTATG
Osteoprotegrin	U94330	TGAGTGTTCTGGTGGACAGTT	ACTGCTTTCACAGAGGTCAATG
GH	NM_001034848	GCGTCTATGAGAAACTGAAGGA	ATGTTGGCGTCAAACTTGTC
PTH	NM_017044	TGATCCTCATGCTGGCAGTT	CCCAGGTTGTGCATAAGCTGTA
Calcitonin	V01228	TTTCCTGGTTGTCAGCATCT	TAGGCGAGCTTCTTCTTCAC
ERα	NM_012689	GGTTGGAGATCCTGATGATTG	CATGCGGAATCGACTTGA
GHR	NM_017094	AGCAGCAAAGGATTAAGATG	AATGCCCAAGATGGTGTT
IGF-1	BC086374	GGTGGACGCTCTTCAGTTC	CCTCCTCAGATCACAGCTC
24, 25-hydroxylase	NM_001108499	TTCCTAAAGGCACAACAGTC	CAAGAACATTTCCATCCGA
25-hydroxylase	NM_178847	GGTCGCAGGATTGCAGAAC	ATGCGGGACACAGACTTCAC
1α-hydroxylase	NM_053763	AACGAAGTTGCATAGGGA	TGTCTACAAACTGGAGATGGAT
24-hydroxylase	NM_201635	CTTCGCTCATCTCCCATT	ATCTCCACAGGTTCATTGTC
Glyceraldehyde-3 phosphate dehydrogenase	NM_017008	AGGTTGTCTCCTGTGACTTC	CTGTTGCTGTAGCCATATTC
Aldehyde dehydrogenase 1 family, member A1 (Aldh1a1)	NM_022407	GTGTGGGTTAACTGCTATATGATCT	AACACTTTGCTGGTGACGAT
Aquaporin 9 (Aqp9)	NM_022960	TCCATTCATATCCACGCC	CCGAGAGAACAGGAAAGGA
Interleukin 1 receptor accessoryprotein (Il1rap)	NM_001167840	ATTTCCGCCTTCCAGAGA	TGGGGGAATTGAAACAGC
Angiopoietin-like 4 (Angptl4)	NM_199115	CCCCACACACCTAGACAATG	CCGCTCCCCTTCTTGAAA
Very low density lipoproteinreceptor (Vldlr)	NM_013155	TCAGTGTATCCCAGAGTCC	CACAAAGTTCCTGGAGACAC
Periostin, osteoblast specific factor (Postn)	NM_001108550	CAACTCCGTGTCTTCGTG	TTCGTGCAGGGACTTCTC
Aggrecan (Acan)	NM_022190	TGATTCTGCCACTGCCTT	TGTGCCTCCTCAAATGTC
Gremlin 1 (Grem1)	NM_019282	CCTGAAGCAGACCATCCAT	GGACAGTTGAGTGTGACCATC
Wingless-type MMTV integration site family, member 10B (Wnt10b)	NM_001108111	ACAGCGCCATCCTCAAG	GCCTGAAGCTGCAACAACT

### Microarray Analysis and Pathway Analysis

The analysis of RNA quality showed that the A260/A280 nm absorbance ratio of RNA samples used in this experiment ranged consistently from 1.8 to 2.0. The qualities of purified RNAs were assessed by an Agilent 2100 Bioanalyzer using an RNA 6000 Nano Kit (Agilent Technologies, Palo Alto, CA, USA). The samples in which the RNA Integrity Number (RIN) score was between 7 to 10 were used in microarray and real-time RT-PCR. An equal amount of RNA from the 3 rats in each group was pooled and used for microarray analysis, as described elsewhere [20], [21]. Briefly, total RNA (100 ng) was reverse-transcribed using a T7 sequence-conjugated oligo dT primer. At the same time, we used an RNA Spike-In Kit One Color (Agilent) to adjust the microarray data. Synthesis, amplification, and labeling of complementary RNA (cRNA) with Cy3 dye were performed according to the manufacturer’s protocols. Prepared cRNA was added to a whole rat genome oligo DNA microarray version 3.0 (4×44 K; Agilent). Hybridization was performed at 65°C for 17 h. After washing, fluorescence intensity was assayed using a scanner (G2565BA; Agilent). The signal intensities of Cy3 were quantified and analyzed by subtracting the background, using Feature Extraction software ver. 10.7.1.1 (Agilent). These data were normalized by GeneSpring GX11.5.1 (Agilent). We selected 16,927 genes having florescence intensities >100 for RNA samples from sham or GX rats using GeneSpring GX11.5.1.

We used Ingenuity Pathway Analysis (IPA; September 2011 version) to determine the functional pathways in the identified genes. IPA software contains a database of biological interactions among genes and proteins, and we used it to calculate the probability of a relationship between each canonical pathway and the identified genes. IPA scans the set of inputs genes to identify networks by using Ingenuity Pathway Knowledge Base (IPKB) for interactions between identified ‘Focus Genes’, (in this study, the differently expressed genes between GX and sham) and known and hypothetical interacting genes stored in the IPA software. The data obtained was used to generate a set of networks with a maximum network size of 35 genes/proteins. Networks are displayed graphically as genes/gene products (‘nodes’) and the biological relationships between the nodes (‘edges’). All edges are from canonical information stored in the IPKB. In addition, IPA computes a score for each network according to the fit of the user’s set of significant genes. The score indicates the likelihood of the Focus Genes in a network from Ingenuity’s knowledge base being found together due to random chance. A score of 3, the cutoff for identifying gene networks, indicates that there is only a 1/1000 chance that the locus genes shown in a network are due to random chance. Therefore, a score of 3 or higher indicates a 99.9% confidence level to exclude random chance.

### Statistical Analysis

Data were shown as mean ± SEM. Statistical analysis was performed by student t-test using StatView software (Abacus Concepts, Berkeley, CA). Differences were considered significant at p<0.05.

## Results

### Body Weights

Body weights at surgery were 303.7±1.7 g in sham (n = 12) and 299.0±2.2 g in GX (n = 16), respectively. There were no significant differences in body weights between sham and GX. Body weights in GX were decreased approximately 10% from the pre-operation level during the first 2 weeks, but were increased in the following 3 weeks. Body weights at sampling were 371.0±10.9 g (n = 12) in sham and 353.5±18.3 g in GX (n = 12), respectively. There were no significant differences in body weights between sham and GX.

### CT-based Assessment of the Femur

**Figure 1:**
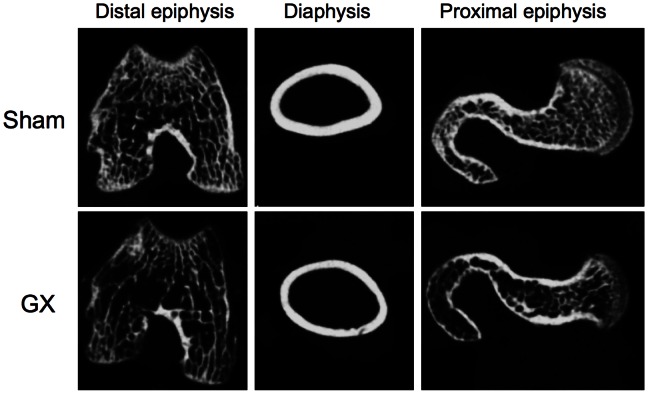
Representative CT images of the femur derived from gastrectomized rats and sham-operated rats. Compared with the sham group (upper column), the cortical bone in the diaphysis is thinner and the cancellous bone in the distal or proximal epiphysis is more scattered in the GX group (lower column).

As shown in Figure 1, the cortical bone was thinner and the cancellous bone was more scattered in GX. The bone mineral contents and the bone mineral densities in the cortical bone and cancellous bone were significantly lower in GX (Table 2). Cortical bone thickness and cortical bone area were also significantly lower in GX (Table 2 and Figure 1). However, there were no significant differences in cancellous bone area and trabecular area between GX and sham. Both the minimum moment of inertia of cross-sectional areas and the polar moment of inertia of cross-sectional areas were also significantly lower in GX (Table 2). As the former value represents the flexural rigidity, and the latter represents the torsional rigidity, the rigidities were severely diminished in GX.

**Table 2:** CT-based bone densitometry of the femur.

	Resorption				Formation			
	ES/BS	Oc.S/BS (%)	N.Oc/BS (N/mm)	BRs.R (mm^2^/mm^2^/year)	OV/BV (%)	OV/OS (mm)	Ob.S/BS (%)	N.Ob/BS (N/mm)
	Eroded surface/Bone surface	Osteoclast surface/Bone surface	Osteoclast number/Bone surface	Bone resorption rate	Osteoid volume/Bone volume	Osteoid volume/Osteoid surface	Osteoblast surface/Bone surface	Osteoblast number/Bone surface
Sham	21.21±2.74	14.5±1.7	4.1±0.5	0.121±0.015	4.18±0.71	4.71±0.29	29.6±4.0	22.2±3.6
GX	36.90±2.19	24.1±0.7	6.4±0.5	0.224±0.016	12.04±0.80	6.96±0.25	44.2±1.4	41.0±1.7
p	p<0.01	p<0.001	p<0.01	p<0.01	p<0.0001	p<0.001	p<0.01	p<0.001
	**Mineralization**				**Bone volume**			
	**MAR (mm/day)**	**BFR/BV (%/year)**	**BFR/BS (%/year)**	**Mlt (day)**	**BV/TV (%)**	**Tb.N (N/mm)**	**Tb.Sp (mm)**	**Tb.Th (mm)**
	**Mineral apposition rate**	**Bone formation rate/Bone volume**	**Bone formation rate/Bone surface**	**Mineralization lag time**	**Bone volume/Tissue volume**	**Trabecular number**	**Trabecular separation**	**Trabecular thickness**
Sham	1.62±0.07	932.0±96.8	0.3±0.04	1.61±0.21	13.0±2.0	2.02±0.30	495.5±89.8	64.9±2.9
GX	2.78±0.09	1976.9±157.1	0.50±0.02	2.25±0.10	5.7±0.9	1.08±0.14	782.4±44.0	51.5±3.3
p	p<0.0001	p<0.001	p<0.001	p<0.05	p<0.01	p<0.05	p<0.05	p<0.05

### Bone Histomorphometry of the Femur and the Calvaria

**Figure 2:**
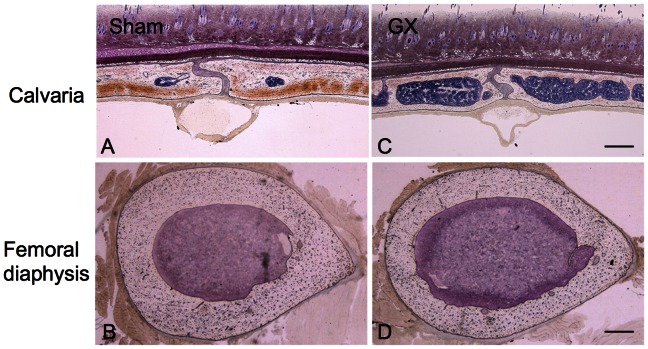
Representative photographs showing horizontal sections of the calvaria and the femoral diaphysis stained with Villanueva bone stain. In GX (C and D), the bone marrow cavities are larger and the cortical bones are thinner in the calvaria (A and C) and in the femoral diaphysis (B and D) compared with sham-operated rats (A and B). Scale bar = 500 µm.

**Figure 3:**
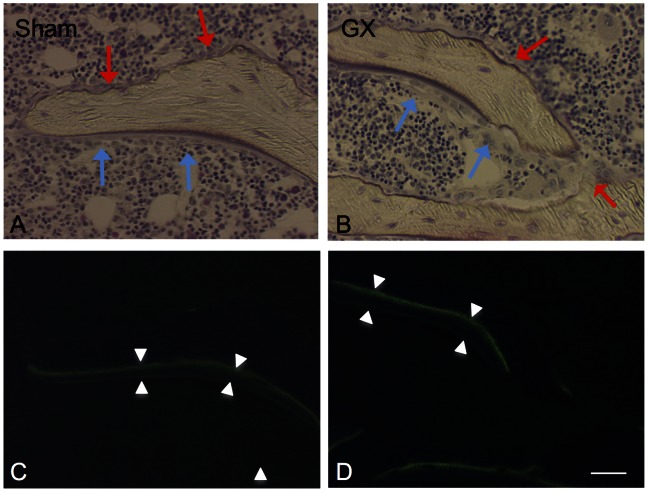
Representative photographs showing microscopic images in the femoral distal epiphysis stained with Villanueva bone stain (A and C) and fluorescence microscopic images of the calcein and tetracycline layers in the same focus plane (B and D). In GX (B) compared with sham (A), the osteoblasts (indicated by blue arrow) are larger in size and increased in number, while the osteoclasts (indicated by red arrow) are also increased in number. The distances between calcein and tetracycline layers as shown by arrow-head were larger in GX (D) compared with sham (B), indicating the mineral apposition rate is increased in GX. Scale bar = 20 µm.

The horizontal sections of the calvaria and the femoral diaphysis stained with Villanueva bone stain are shown in Figure 2. In GX, there were larger bone marrow cavities and thinner bones in the calvaria and the femoral diaphysis. Microscopic images of the femoral distal epiphysis stained with Villanueva bone stain and fluorescence microscopic images of the calcein and tetracycline layers are shown in Figure 3. In GX, the osteoblasts were larger in size and increased in number, and the osteoclasts were also increased in number. The distances between the calcein and tetracycline layers, which reflect the mineral apposition rate, were larger in GX. In addition, the layer of osteoid was thicker in GX.

The values for the histomorphometric parameters are presented in Table 3. First, parameters for bone resorption such as eroded surface/bone surface (ES/BS), osteoclast surface/bone surface (Oc.S/BS), osteoclast number/bone surface (N.Oc/BS) and bone resorption rate (BRs.R) were increased significantly in GX. Second, parameters for bone formation such as osteoid volume/bone volume (OV/BV), osteoid volume/osteoid surface (OV/OS), osteoblast surface/bone surface (Ob.S/BS) and osteoblast number/bone surface (N.Ob/BS) showed significant increases in GX. Notably, OV/BV was three times higher in GX than that in sham. Third, parameters for mineralization such as mineral apposition rate (MAR), bone formation rate/bone volume (BFR/BV), bone formation rate/bone surface (BFR/BS) and mineralization lag time (Mlt) were significantly higher in GX. Finally, parameters for bone volume such as bone volume/tissue volume (BV/TV), trabecular number (Tb.N), trabecular separation (Tb.Sp) and trabecular thickness (Tb.Th) were significantly lower in GX.

**Table 3:** The histomorphometric parameters in the distal epiphysis of the femur.

	cortical bonemineral density(mg/cm^3^)	cancellous bone mineral density(mg/cm^3^)	cortical bone mineral content (mg)	cancellous bone mineral content (mg)	cortical bone thickness (cm)	cortical bone area (mm^2^)	cancellous bone area (mm^2^)	trabecular area (mm^2^)	minimum moment of inertia of cross-sectional area (mg⋅cm)	polar moment of inertia of cross-sectional area (mg⋅cm)
Sham	1122.1±13.3	506.2±11.5	234.1±10.4	196.6±5.8	0.037±0.001	0.054±0.001	0.101±0.004	0.071±0.003	1.626±0.046	4.954±0.202
GX	1035.0±7.6	373.9±8.0	180.0±3.5	160.9±6.7	0.030±0.001	0.045±0.001	0.112±0.003	0.067±0.003	1.315±0.046	3.932±0.135
P	p<0.001	p<0.0001	p<0.001	p<0.01	p<0.001	p<0.001	NS	NS	p<0.001	p<0.01

### Serum Biochemical Parameters

Serum concentrations of factors affecting Ca and bone metabolism are presented in Table 4. Serum Ca concentration was slightly but significantly decreased, while serum iP concentration was significantly increased in GX. The concentration of 1, 25(OH)_2_D_3_, the active form of VD was significantly increased 7-fold in GX over that of sham, while that of 25(OH)D_3_, the predominant circulating form of VD, was reduced significantly in GX. The concentrations of PTH and calcitonin were not significantly different between GX and sham-operated rats.

**Table 4:** The biochemical parameters in the serum.

	Ca (mg/dl)	iP (mg/dl)	1,25(OH)_2_D_3_ (pg/ml)	25(OH)D_3_ (ng/ml)	PTH (pg/ml)	calcitonin (pg/ml)
Sham	10.0±0.1	7.7±0.4	102.9±13.0	35.7±2.2	28.2±4.7	85.1±3.9
GX	9.6±0.1	9.3±0.3	756.4±42.2	17.6±1.3	32.0±6.2	77.4±1.1
p	p<0.05	p<0.01	p<0.0001	p<0.0001	NS	NS

### Expression of mRNAs in the Bone, Liver, Kidney, Pituitary Gland and Thyroid

Expression of mRNAs affecting Ca and bone metabolism in the bone, liver, kidney, pituitary gland and thyroid is presented in Table 5. The mRNA levels of osteoclast-derived tartrate-resistant acid phosphatase 5b (TRACP-5b) in the bone were significantly increased in GX, while those of osteocalcin, produced in the mature osteoblasts, were also increased significantly in GX compared to sham-operated rats. The mRNA levels of receptor activator of nuclear factor-κB (RANK) and osteoprotegerin (OPG) were not significantly different between sham and GX, while those of RANK ligand (RANKL) were increased in GX significantly. Among the enzymes involved in VD biosynthesis in the liver and kidney, the mRNA levels of 1α-hydroxylase in the kidney were increased significantly in GX, while those of other enzymes such as 24, 25-hydroxylase, 25-hydroxylase and 24-hydroxylase were not significantly different between sham and GX. The mRNA levels of PTH in the thyroid were significantly reduced in GX, while those of calcitonin were not significantly different from sham-operated rats. In the liver, those of estrogen receptor (ER) α and growth hormone receptor (GHR) were significantly reduced in GX compared with sham-operated rats, whereas those of insulin-like growth hormone (IGF)-1 in the liver and growth hormone (GH) in the pituitary gland were not significantly different.

**Table 5:** Fold changes in the gene expressions in the bone, liver, kidney, pituitary gland and thyroid.

Bone					
	Osteoclast-derived Tartrate-resistant Acid phosphatase 5b	Osteocalcin	RANK	RANKL	Osteoprotegrin
Sham	100±24.6	100±13.9	100±26.4	100±17.8	100±18.4
GX	330.0±37.7	252.0±60.7	83.8±19.0	185.7±24.8	84.9±17.0
p	p<0.001	p<0.05	NS	p<0.05	NS
Liver					
	ERα	GHR	IGF-1	24, 25-hydroxylase	25-hydroxylase
Sham	100±15.8	100±6.1	100±13.0	100±7.7	100±13.5
GX	56.7±7.6	68.2±6.8	97.8±11.9	99.3±11.3	94.8±5.8
p	p<0.05	p<0.01	NS	NS	NS
Kidney					
	24, 25-hydroxylase	1α-hydroxylase	24-hydroxylase		
Sham	100±22.2	100±14.3	100±26.7		
GX	78.9±8.3	1248±304	50.8±13.4		
p	NS	p<0.01	NS		
Pituitary gland		Thyroid			
	GH		PTH	Calcitonin	
Sham	100±3.1	Sham	100±8.8	100±23.9	
GX	109.0±6.4	GX	65.0±12.6	175.7±110.4	
p	NS	p	p<0.05	NS	

### Microarray Analysis and Data Mining by IPA

We investigated the gene expression profiles of the bone in response to GX using microarray analysis. Among 30,367 genes that were analyzed, 16,927 genes were detected in the GX and/or sham-operated rats. We selected genes whose expression differed by more than 2-fold in GX compared with sham. Using these criteria, 612 genes were up-regulated and 1,097 genes were down-regulated in GX compared with sham-operated rats.

**Figure 4:**
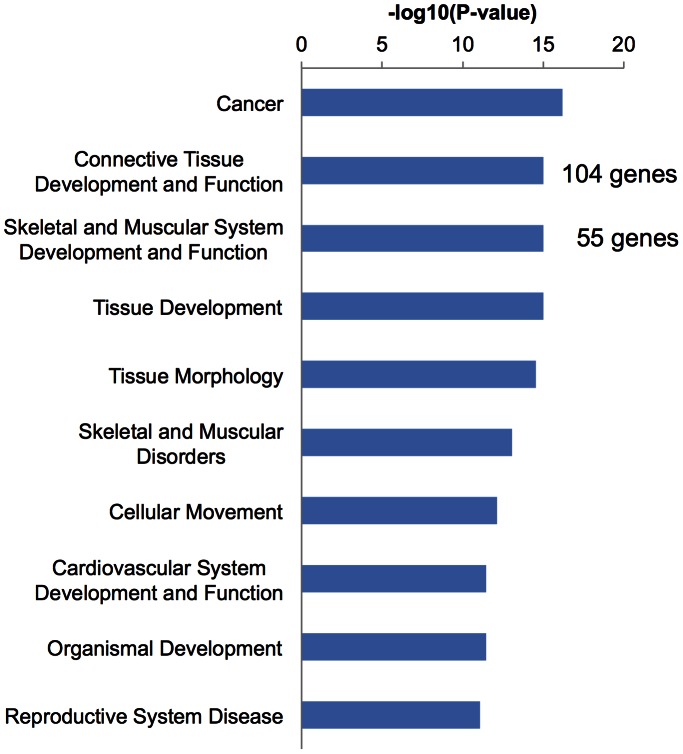
Microarray analysis and data mining using IPA-pathway analysis (biological function). IPA was used to organize the differentially expressed genes into functionally annotated pathways and networks. In biological functions modified significantly by the 1,709 genes whose expression changed by GX, connective tissue development and function (p = 9.67E-10) and skeletal and muscular system development and function (p = 9.67E-10) were listed. A total of 104 genes were included in connective tissue development and function, and 55 genes were in skeletal and muscular system development and function.

**Figure 5:**
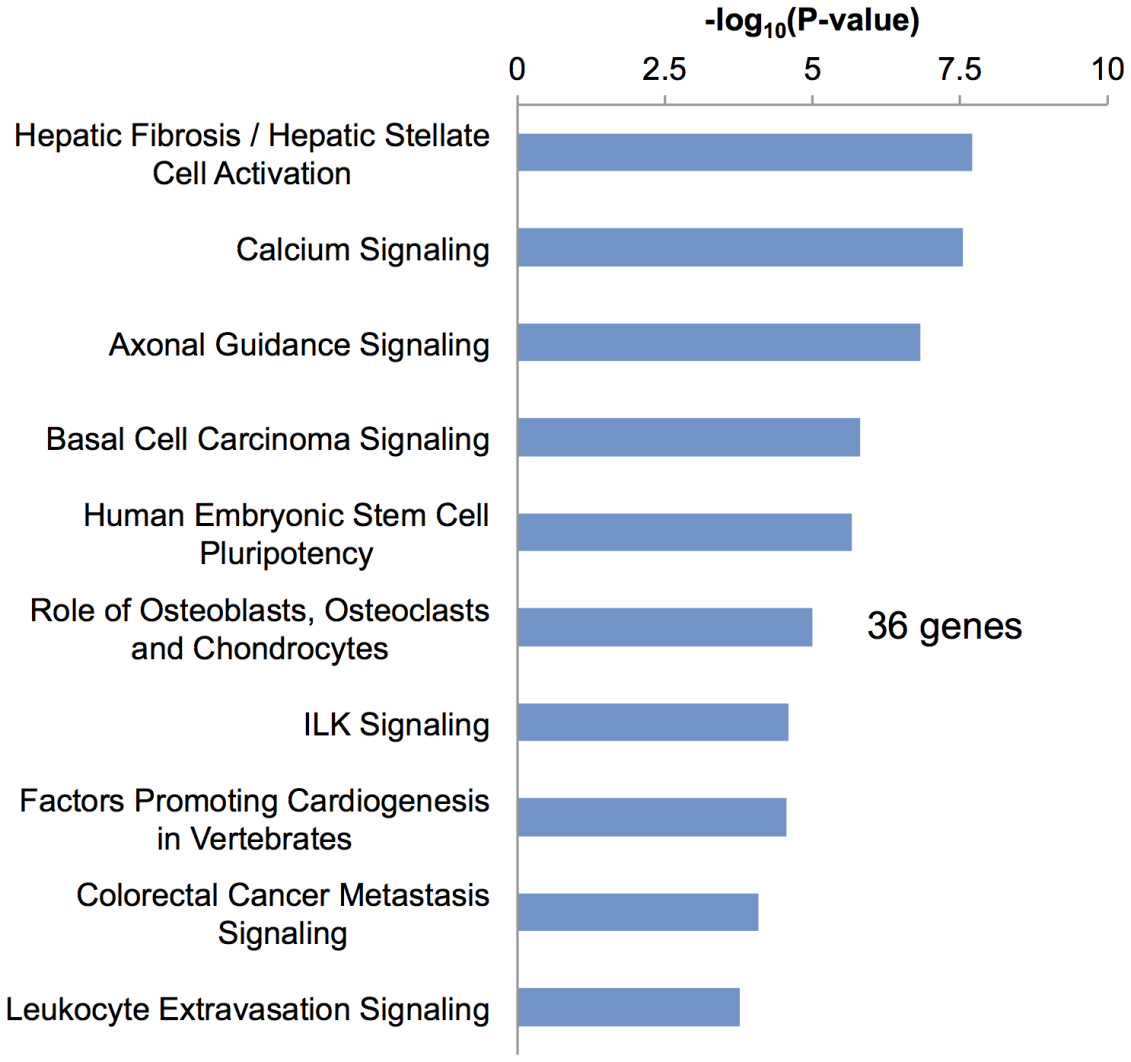
Microarray analysis and data mining using IPA-pathway analysis (canonical signal pathways). In canonical signal pathways, the role of osteoblasts, osteoclasts and chondrocytes (p = 5.00E-3) was listed. A total of 36 genes were included in this pathway.

**Figure 6:**
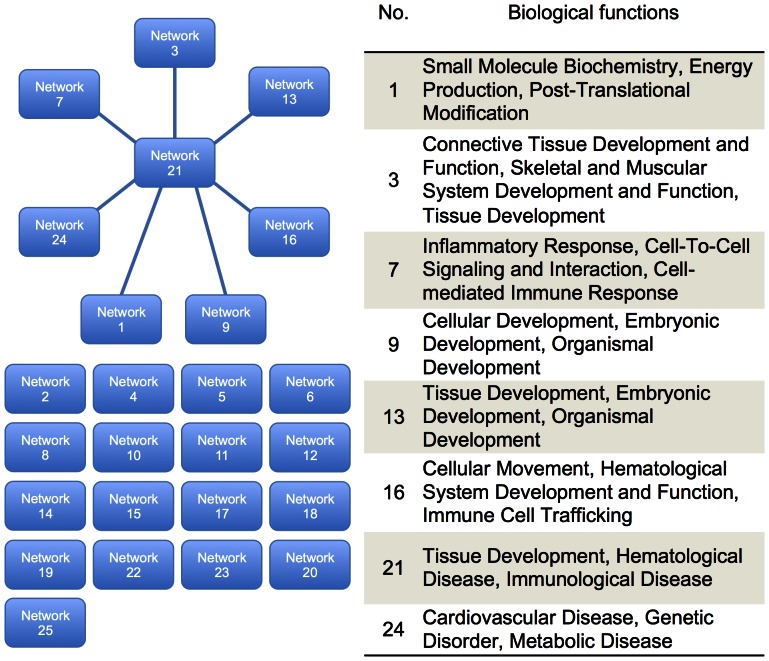
Network analysis to identify novel mechanisms related to the effect of GX. Twenty-five networks were generated from the 1,709 genes. Eight networks formed hub constructions. Network 21 was the central hub, whose biological functions were tissue development, hematological disease and immunological disease.

IPA was used to organize the differentially expressed genes into functionally annotated pathways and networks. Using IPA, we identified canonical pathways and biological functions modified significantly by the 1,709 genes whose expression changed following GX. Among the identified biological functions, connective tissue development and function (p = 9.67E-10), and skeletal and muscular system development and function (p = 9.67E-10) were listed as shown in Figure 4. Among the identified canonical pathways, the role of osteoblasts, osteoclasts and chondrocytes (p = 5.00E-3) was observed as shown in Figure 5. Furthermore, in a search for novel mechanisms related to the effects of GX, we performed network analysis (Figure 6). IPA generated 25 networks from the 1,709 genes, and 7 networks had 1 or 2 genes included in 1 network. Thus, we focused on these 8 networks that formed a hub construction. Network 21 was the central network, whose biological functions were tissue development, hematological disease and immunological disease. The 9 genes that belonged to 2 networks were included as hub genes (Table 6). To confirm these networks, the expression of these 9 genes was evaluated by real-time RT-PCR. The mRNA levels of aldehyde dehydrogenase 1 family, member A1 (Aldh1a1), interleukin 1 receptor accessory protein (Il1rap), very low density lipoprotein receptor (Vldlr), periostin (Postn), aggrecan (Acan), gremlin 1 (Grem1) and wingless-type MMTV integration site family, member 10B (Wnt10b) were reduced significantly, while those of angiopoietin-like 4 (Angptl4) were increased significantly. Expression levels of aquaporin 9 (Aqp9) were high in GX without significance.

**Table 6:** Validation of gene expression changes in the bone.

		Sham	GX	p	Network
Aldehyde dehydrogenase 1 family, member A1 (Aldh1a1)		100±20.5	37.1±7.5	p<0.05	21, 1
Aquaporin 9 (Aqp9)		100±16.2	155.4±40.4	NS	21, 7
Interleukin 1 receptor accessory protein (Il1rap)		100±11.9	46.9±11.9	p<0.05	21, 7
Angiopoietin-like 4 (Angptl4)		100±38.5	672.2±131.5	p<0.01	21, 24
Very low density lipoprotein receptor (Vldlr)		100±15.4	42.7±15.0	p<0.05	21, 24
Periostin, osteoblast specific factor (Postn)		100±24.7	33.5±77.7	p<0.05	21, 16
Aggrecan (Acan)		100±20.4	59.6±5.3	p<0.05	21, 13
Gremlin 1 (Grem1)		100±13.7	47.0±10.9	p<0.05	21, 9
Wingless-type MMTV integration site family, member 10B (Wnt10b)		100±13.3	60.5±7.5	p<0.05	21, 3

## Discussion

GX has been described to result in osteomalacia due to deficiencies in VD and Ca. In our study, detailed histological and biochemical examinations demonstrated that this was not the case. Further, we propose that GX-induced pathological changes in bone might reflect a partial manifestation of chronic systemic inflammation.

X-ray CT data indicated significantly reduced bone mineral content and bone mineral density in the cortical bone and cancellous bone in GX (Figure 1 and Table 2). This data suggests that GX-induced bone changes are compatible with osteomalacia. However, the X-ray CT findings indicate a static state in the bone, not dynamic bone turnover. Thus, the decrease in bone mineral content may not always reflect a decrease in dynamic mineralization.

The bone histomorphometric study indicated that bone resorption, bone formation and mineralization were increased significantly and bone volume was decreased significantly (Figures 2, 3 and Table 3). Therefore, GX-induced bone changes indicate a high turnover rate in bone, and an increased coupling of resorption and synthesis in bone remodeling. It is noted that OV/BV was three times higher in GX, indicating a prominent accumulation of osteoid. Both MAR, BFR/BV and Mlt were also significantly increased. This can be considered a result of formation of osteoid, which was increased substantially, while mineralization was comparatively delayed or did not catch up with the increased formation of osteoid. Another interpretation is that the proportion of osteoid was increased since osteoclasts resorb the calcified bone but not osteoid. Histomorphometrically, this condition can be considered to exhibit a high turnover rate in bone with hyperosteoidosis and increased mineralization. The primary mineralization is that of osteoid, while the secondary mineralization is the long-lasting maturation of the bone. In high turnover bone, the primary mineralized bone is rapidly resorbed before the secondary mineralization, resulting in an immature and poorly mineralized bone [22]. Thus, the CT finding does not conflict with the results in the bone histomorphometry.

Osteomalacia is characterized by an impairment of bone mineralization with an accumulation of osteoid [18]. GX-induced bone changes do not accord with the criterion of osteomalacia since MAR, BFR/BV and BFR/BS were increased significantly in GX although primarily as an accumulation of osteoid. In most cases, osteomalacia is caused by a decrease in circulating Ca versus phosphate product [18]. The other previous biochemical observations demonstrated the absence of intestinal Ca mal-absorption, VD, Ca deficiency in FX or GX rats [4]–[6], [9]. Therefore, GX-induced bone changes may be fundamentally different from typical osteomalacia due to deficiencies in VD and Ca. Ovariectomy (OVX)-induced bone loss is a similar high turnover in bone. However, GX-induced bone changes seem to be different from OVX-induced bone loss since OV/BV is prominently increased in GX, while it is not changed in OVX [23]. Primary hyperparathyroidism is characterized by rapid remodeling. However, the balance between resorption and formation in the cancellous compartment is conserved since cancellous bone volume is normal [24]. Metabolic bone diseases frequently seen in chronic kidney disease patients are secondary hyperparathyroidism, osteomalacia, aluminum bone disease, adynamic bone disease and mixed uremic osteodystrophy [25]. In secondary hyperparathyroidism, serum PTH levels are higher than those in primary hyperparathyroidism. Secondary hyperparathyroidism displays a more prominent increase in remodeling parameters, accompanied by depositions of woven osteoid and variable amounts of peritrabecular marrow fibrosis. However, peritrabecular marrow fibrosis was never observed in GX. In addition, serum PTH levels were not increased in GX, consistent with results of a previous study [9]. Therefore, high turnover in bone with hyperosteoidosis and increased mineralization as observed in GX is quite unique. In addition, the occurrence where the volume of new osteoid formation exceeds that of mineralization is often observed in patients with chronic kidney disease suffering from mixed osteodystrophy caused by secondary hyperparathyroidism. In these patients, bone resorption, osteoid formation and mineralization are often increased. However, mineralized bone volume decreases if bone resorption exceeds mineralization in these patients [26]. Similar findings were observed in the present study.

As shown in Table 4, slightly decreased serum Ca levels and slightly increased serum iP levels suggest that Ca metabolism is abnormal in GX, although in previous GX or FX studies serum mineral levels were normal [6], [9]. Prominent increased serum levels in 1, 25(OH)_2_D_3_, decreased serum levels in 25(OH)D_3_ and normal serum levels in PTH and calcitonin are consistent with results of previous studies [6], [9]. These results indicate that conventional bone and Ca metabolic pathways are not involved or changed in compensation.

Expression of mRNA closely related with bone and Ca metabolism in the bone and other organs was also investigated (Table 5). In bone, the expression of TRACP-5b, a marker of bone resorption [27], was increased significantly in GX, while that of osteocalcin, a marker of bone formation [28], was also increased significantly in GX compared with sham-operated rats. These results suggest high turnover of bone, as shown in the bone histomorphometric study. The RANK/RANKL/OPG pathway regulates the balance between the activity of osteoclasts and osteoblasts in bone remodeling [29]. RANKL, a member of the TNF ligand family is produced by osteoblast lineage cells and activated T-cells. Macrophage colony-stimulating factor (M-CSF) increases the pool of osteoclast precursors, whereas RANKL binds to its receptor RANK on osteoclast precursors and mature osteoclasts, and enhances osteoclast differentiation. OPG acts as a decoy receptor to RANKL and inhibits osteoclast activation and bone resorption. Expression of mRNA levels of RANK and OPG did not differ between GX and sham-operated rats, suggesting that an involvement of the RANK/RANKL/OPG pathway may be limited in GX-induced bone pathology. However, as the mRNA level of RANKL was increased significantly in GX, it may contribute to bone resorption.

The mRNA levels of 1α-hydroxylase in the kidney were increased significantly in GX in accordance with the increase in serum 1, 25(OH)_2_D_3_ and decrease in serum 25(OH)D_3_. However, the supraphysiological level of 1, 25(OH)_2_D_3_ stimulates bone resorption through the expression of RANKL in vivo. Therefore, bone resorption may be aggravated further due to increased 1, 25(OH)_2_D_3_ in GX, resulting in the reduction of both bone mineral density and bone mineral content although mineralization parameters were increased [30]. These changes may compensate for the decrease in bone mineral contents and serum Ca levels if the serum level of 1, 25(OH)_2_D_3_ was within the normal range. Bone formation without prior bone resorption, or “minimodeling”, compensates for bone loss in both cancellous bone and cortical bone following suitable exercise [31], [32].

Parietal cells and basal granulated cells in gastric mucosa produce and secrete estrogen [33] and ghrelin [34], respectively. Reduction of portal venous estrogen levels in GX results in a decrease in ERα mRNA levels, consistent with our previous observation [33]. Gastric estrogen is trapped by hepatic ERα, and it does not overflow into systemic circulation, suggesting that gastric estrogen is not directly involved in bone metabolism [33]. Ghrelin stimulates the secretion of GH in the pituitary. GH and insulin-like growth factor 1 (IGF-1) produced in the liver under GH control are involved in bone metabolism [35]. IGF-1 enhances the differentiated function of osteoblasts and bone formation. Adult GH deficiency causes low bone turnover. In the present study, GH and IGF-1 were not involved since GX-induced bone changes include high bone turnover. In fact, mRNA expression of GH in the pituitary and that of IGF-1 in the liver were not significantly different between GX and sham-operated rats, although we did not estimate the serum GH and IGF-1 levels in this study. As serum ghrelin levels are decreased in FX [8] and ghrelin stimulates the proliferation of osteoblasts in vitro [36], reduction of ghrelin may be involved in GX-induced bone changes. However, this is also not likely since bone formation was enhanced in GX. In fact, knockout studies indicate that ghrelin is not critically for bone density [37].

Since these conventional factors could not account for the GX-induced high turnover in bone with hyperosteoidosis and increased mineralization, we performed transcriptosome analysis and pathway analysis in bone.

Microarray analysis indicated that 612 genes were up-regulated and 1,097 genes were down-regulated in GX. In order to organize the differentially expressed genes into functionally annotated pathways and networks, IPA was applied. Among the biological functions, connective tissue development and function, and skeletal and muscular system development and function were indicated as shown in Figure 4. In canonical pathways, the role of osteoblasts, osteoclasts and chondrocytes was indicated as shown in Figure 5. Furthermore, we performed network analysis to reveal novel mechanisms related to the effect of GX. IPA generated 25 networks from the 1,709 genes, and 8 networks formed hub constructions (Figure 6). The center was Network 21, whose biological functions were tissue development, hematological disease and immunological disease. To confirm these connections, we evaluated the expression of each hub gene. All genes except one were significantly increased or decreased in GX (Table 6), suggesting these networks were almost valid. In this biggest network, inflammatory response, cell-mediated immune response, immune cell trafficking and immunological disease were included as main biological functions. Biological functions related to development were also characteristic.

Several genes in this hub are involved in bone metabolism. Periostin (Postn) is a matricellular glutamate-containing protein expressed in adult connective tissues under mechanical stress, including the periosteum [38]. Gremlin (Grem1) is a secreted glycoprotein that antagonizes the action of bone morphogenic proteins-2, -4 and -7 [39]. Aggrecan (Acan) is the cartilage specific proteoglycan and a main compartment of the extracellular matrix [40]. Activation of Wingless-type MMTV integration site family (Wnt) signaling is associated with expansion of the osteoblast and chondrocyte lineages in vivo and in vitro. Wnt10b-null mice reduce trabecular bone and osteoblast differentiation markers [41]. Angiopoietin-like 4 (Angptl4) stimulates the osteoclast resorptive activity in a hypoxia-inducible factor-1a-dependent manner [42]. Aquaporin 9 (Aqp9) mediates the passage of a wide variety of non-charged solutes in addition to water, is expressed in osteoclast-lineage cells, and is involved in the fusion process [43]. However, the precise contributions of these genes to GX-induced high turnover bone with hyperosteoidosis and increase of mineralization are unclear.

Intestinal microflora is involved in trophic effects on intestinal epithelia and on the immune system, and in protective functions against alien microbes [44]. Reduction of the gastric acid barrier by Helicobacter pylori-induced chronic atrophic gastritis, long-term use of PPI and GX leads to an alteration in lower intestinal microflora. Recently, in rat treated with omeprazole (100 mg/kg BW/day), we demonstrated a significant increase in the *Lactobacilli group* and *Veillonella*, which are anaerobic bacteria of oropharyngeal origin, and a marked increase in the *C. coccoides* group, *Prevotella*, and especially the *B. fragiltis* group, which has been implicated in colonic carcinogenesis [45]. Furthermore, we have observed a prominent infiltration of inflammatory cells as well as a significant alteration of inflammatory-related genes in the mucosa and submucosa of the colon following GX (unpublished observation). Relations between the alteration of intestinal microflora and chronic systemic inflammation including bone, digestive and other organs are under investigation by our group.

In conclusion, GX resulted in high turnover in bone with hyperosteoidosis and increased mineralization compared with sham-operated rats. Several sets of functionally related genes including those involved in inflammatory responses and development, but not conventional factors are involved in this unique bone pathology. Although the precise mechanisms are not clarified, chronic systemic inflammation may underlie the GX-induced pathological changes in bone.
